# Consumption of indigenous medicines by pregnant women in North India for selecting sex of the foetus: what can it lead to?

**DOI:** 10.1186/s12884-015-0647-4

**Published:** 2015-09-04

**Authors:** Sutapa Bandyopadhyay Neogi, Preeti H. Negandhi, Abhijit Ganguli, Sapna Chopra, Navraj Sandhu, Ravi Kant Gupta, Sanjay Zodpey, Amarjeet Singh, Arun Singh, Rakesh Gupta

**Affiliations:** Indian Institute of Public Health-Delhi (IIPH-D), Public Health Foundation of India (PHFI), New Delhi, India; Thapar University, Patiala, India; Government of Haryana, Chandigarh, India; National Health Mission, Panchkula, Haryana India; Department of Community Medicine, Post Graduate Institute of Medical Education and Research, Chandigarh, India; Rashtriya Bal Suraksha Karyakram, Government of India, New Delhi, India

## Abstract

**Background:**

Sex ratio is an important indicator of development. Despite all the measures undertaken for improvement, it remains an issue of concern in India, with Haryana having a very low sex ratio in the country. Studies have been conducted indicating that consumption of indigenous drugs used for sex selection (SSD) could be strongly associated with adverse effects on the foetal development, including congenital malformations. Some samples of SSDs were collected from parts of North India and analysed in a standard laboratory for its components.

**Methods:**

Thirty SSDs used by the local community were procured from various sources in north India through a rigorous process of collection. These were subjected to laboratory tests to investigate the presence of phytoestrogen and testosterone. Following sample extraction, thin layer chromatography and high performance liquid chromatography were carried out for analysing phytoestrogen content.

**Results:**

SSDs were available in various forms such as powder, tablets, mostly from faith healers. Around 87 % of the samples collected from sources like doctors, quacks and faith healers were to be taken by the pregnant women after conception; 63 % drugs were strongly positive for phytoestrogens (genistein, daidzein, formononetin) and 20 % drugs were positive for testosterone. The average dose of the components as calculated after analyses was as follows: daidzein - 14.1 mg/g sample, genistein - 8.6 mg/g sample, formononetin - 5 mg/g sample.

**Conclusion:**

These SSDs could be potentially detrimental to the growth and development of the foetus. This is likely to have implications on the health of the community. In view of the results obtained in our study, we strongly attest the importance in curbing this harmful practice by banning the supply of the drugs as well as by advocating behavioural changes in the community.

## Background

Sex ratio has been an important indicator of development since decades. Census data for the past 100 years have shown a skewed pattern of the sex ratio in favour of males. Traditionally, it has been observed that there is a preference for sons in India. Various means to have a son include going through multiple pregnancies till a son is born, sex selective abortions, pre-conceptional techniques and post-conceptional intake of drugs to beget a male child. This has nation-wide implications, especially for issues such as female foeticide and health care for the living girls. The PNDT Act (Pre Natal Diagnostic Technique) brought into force since 1994 banned the detection of sex of the foetus and has been a useful step towards curbing female foeticide. A subsequent amendment in 2002 brought pre-conceptional sex selection techniques also into its fold since people were resorting to pre-conceptional methods, thereby changing the PNDT Act to PCPNDT Act (Pre Conception, Pre Natal Diagnostic Technique). Despite all these measures, sex ratio continues to be a concern. Haryana, an Indian state, has one of the lowest sex ratios (879 females per 1000 males) in comparison to the national figure of 933 females per 1000 males [1, 2].

Besides sex selective abortions, several indigenous methods are in vogue for giving birth to a male child. Some of these include consumption of special diet before and after conception, timing and date of sexual intercourse (even dates such as 2nd, 4th, 6th of the month for male offspring or full moon/no moon) and reciting chants. The rituals adopted for conception of healthy male child is described as *“Pumsavana karma.”* Intake of indigenous medicines for sex selection in India as well as the West is also documented [3, 4]. Anecdotal reports and research findings highlighting this practice have found their place in newspapers over the last two decades. In 1991, Government of Gujarat, India, banned the manufacture and sale of *Select*, a drug which was claimed to produce a male foetus if a pregnant woman consumed it for 45 days after her last menstrual period [5].

Consumption of indigenous medicines used for sex selection, also known as Sex Selection Drugs (SSD) during the first trimester of pregnancy is prevalent in certain parts of the country [3, 6, 7]. A case—control study was undertaken in the state of Haryana, India recently to investigate the risk factors for congenital malformations. Data regarding congenital malformations and the risk factors were collected from across all 21 districts of the state. Factors such as having more than two living children and the intake of sex selection drugs were found to be strongly associated with congenital malformations [8]. Similar reports were observed in an earlier hospital-based study [9]. It is important to note that SSDs are consumed during the most critical period of development of the embryo. To probe further into the details of the drugs consumed, some samples of SSDs were collected from various parts of North India and laboratory analyses were carried out to investigate the presence of certain components which might have an effect on the development of the embryo. The results of the analyses are presented here.

## Methods

This study was undertaken as a part of a larger case—control study on “Risk factors of congenital malformations in Haryana” described elsewhere [8].

### Collection of SSDs

In order to get information about the drugs, understand the methods of procuring them as well as collecting the drugs for analyses, the research team spoke to women who had delivered and also various genres of people including rickshaw pullers, drivers, cobblers and commoners in different areas of Haryana. Confidentiality was maintained throughout the study. It was indeed a tough task for the team to extract information about the suppliers of these drugs, since people maintained secrecy on this sensitive issue. It was observed that asking direct questions to the respondents would not help in extracting correct information, so information was gathered in a more informal and indirect manner. Finally, based on the information gained thus, the team managed to procure samples from various districts across the states of North India. Samples were collected and stored in polypropylene bottles in moisture-free conditions and transferred to the laboratory at Department of Biotechnology, Thapar University, Patiala for analyses within 17–24 h. They were stored in dark at 28 °C.

### Biochemical analysis of SSDs

A previous study had indicated that the SSDs contain steroids and testosterone [4]. The focus in the current research for laboratory investigations was restricted to testosterone and phytoestrogens in the drugs, since these are known to be the most commonly associated components to affect the development of the embryo. Owing to the high cost of the test kits for testosterone, this component was not tested in all of the procured samples. Phytoestrogen, however, was tested in all the procured samples. The following methods were used for analyses:

1. Sample extraction was done as proposed by Beck in 1964 [10]. All extracts were coded and analysed further.
2. Thin layer chromatography (TLC): The solvent system used was Chloroform/methanol (89: 11, v/v) on silica gel plates. All phytoestrogen standards (Sigma, Mo USA) could be visualized as blue-white fluorescent spots under UV light (257 nm). The experiment was repeated at least thrice to confirm initial authentication.
3. Identification and final quantitation High performance liquid chromatography (HPLC): All HPLC analyses were performed using a Reverse phase column (C-18) octadecylsilane (4 mm I.D × 30 cm). Detector used was UV–VIS variable wavelength detector. Column temperature was maintained at 30 °C. Detection of phytoestrogens was made at 254 nm (optical bandwidth 8 nm).

Separation was achieved by using a linear methanol—water gradient system at a flow rate of 1.0 ml/min. Methanol and water reservoirs each contained 1 % glacial acetic acid (v/v) and 0.01 M ammonium acetate HPLC grade by Casteele et al. [11].

The gradient was programmed to increase from 53 to 58 % reservoir B (methanol) over 30 min. Preliminary peak identification was based on a comparison of retention times of phytoestrogens standards and unknown peaks in the sample extracts.

The preliminary characterization of phytoesterogens by TLC in the drug samples was confirmed by HPLC analysis. A quantitation from HPLC peak area indicated the approximate quantities of each phytoestrogen. The results are presented as the proportion of samples detected positive for phytoestrogens and testosterone. Samples found strongly positive for these ingredients were subjected to further analyses and the average doses of each phytoestrogen consumed were calculated. Quantification of testosterone was however, not done.

### Ethical considerations

The study was approved by Institutional Ethics Committee of Indian Institute of Public Health, Delhi. The participants of the study were mostly women who had delivered. Written consent was taken from them before interviewing after explaining to them about the study.

## Results

A total of 30 samples were collected from various sources ranging from doctors/quacks to faith healers in North India.

The sources provided instructions for varied methods of consumption of the drugs. One such instruction included the consumption of this drug with cow’s milk, to be consumed early morning without looking at any other female.

These drugs were given to the pregnant lady in specific doses to be consumed during the first trimester after confirmation of pregnancy, particularly during 6–10 weeks of pregnancy. They were available in different forms for consumption. Some drugs were to be taken in the form of a powder; some were in the form of tablets. In some cases, fruits injected with some medicines were also given. Some even gave a piece of paper with text written on it in a particular kind of ink to the pregnant lady for consumption. This paper was to be consumed with cow’s milk. These drugs, famous for ensuring the birth of a male child upon consumption, are known locally as “*sau badalne ki dawai*”. Some of these are available at a chemist’s or a grocer’s shop, with faith healers and local villagers. *Rithaphal*, *Majuphal*, *Shivalingi* are some of the common ingredients. These drugs are usually to be taken for 3–5 days or sometimes even for 21 days.

**Fig. 1:**
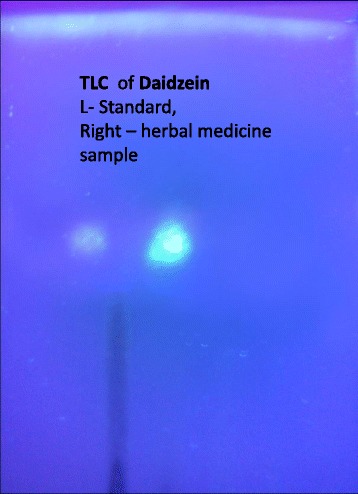
Thin layer chromatography Spot of daidzein extracted from sex selection drugs

**Fig. 2:**
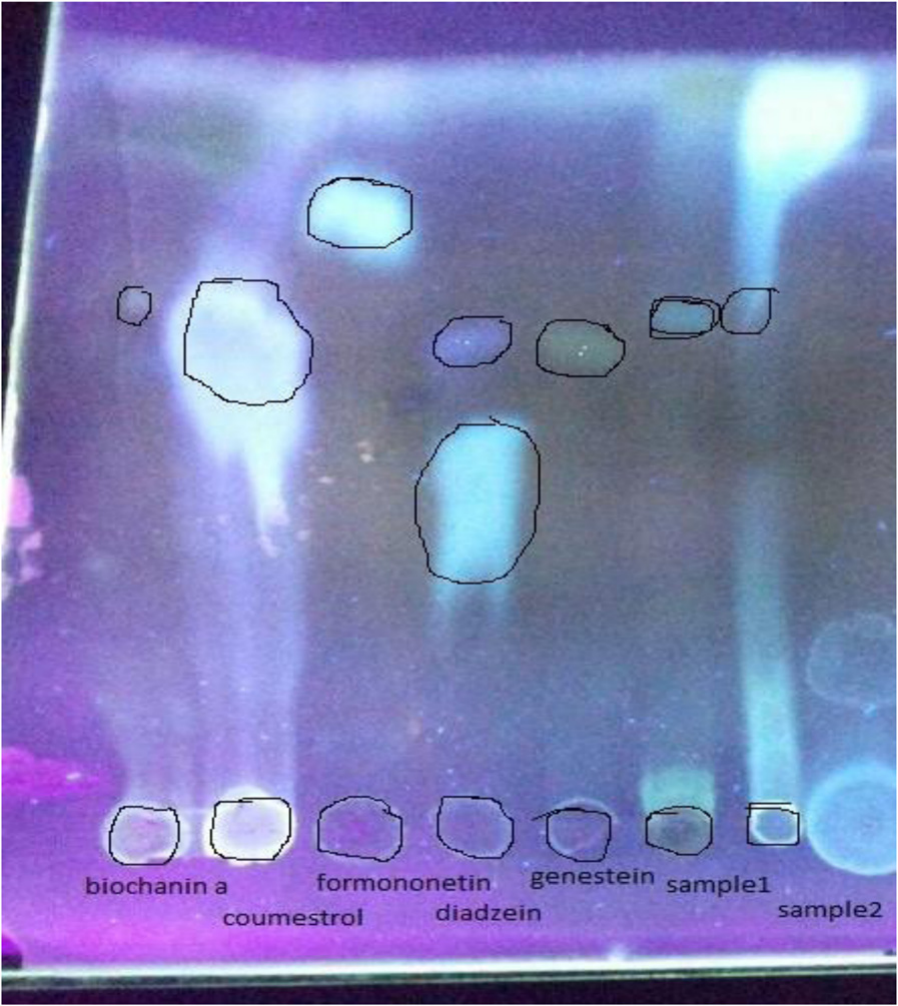
Thin layer chromatography Spot of different phytoestrogens extracted from sex selection drugs

Out of the 30 samples collected, 26 (87 %) were supposed to be taken by women post-conception, three were to be consumed by males pre-conception and one by both the partners prior to conception. Nineteen samples (63 %) were strongly positive for all phytoestrogens (genistein, daidzein, formononetin). (Figures 1, 2) Testosterone was detected in three out of the 15 samples analysed for testosterone.

**Fig. 3:**
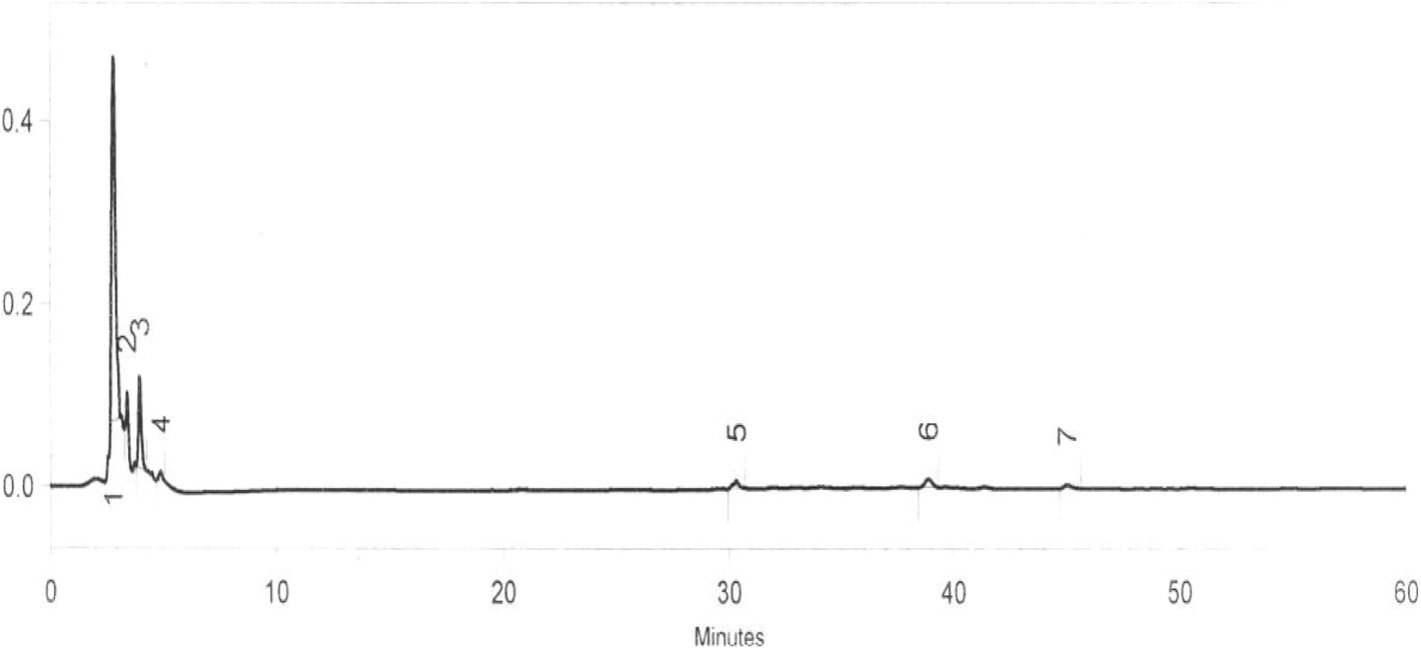
HPLC chromatogram of daidzein extracted from sex selection drugs

Quantitation of each phytoestrogen present was analysed from HPLC reports (Fig. 3, Table 1) An average dose was calculated thereafter based on this data and were as follows: daidzein: 14.10 mg/g sample, genistein: 8.52 mg/g sample, formononetin: 5.09 mg/g sample. The average weight of each sample was 10 g to be consumed over a period of few days ranging from 3 to 21 days and hence daily dose was difficult to calculate. However, the total quantity to be consumed represents a ten-fold increase over that recommended for dietary intake [12, 13].

**Table 1:** Concentration of each phytoestrogen in samples of sex selection drugs

Sample no	Daidzein (mg/g)	Genistein (mg/g)	Formonetin (mg/g)	Testosterone
1	1.00	8.80	6.00	Not done
2	13.00	8.70	5.00	Not done
3	13.00	8.50	5.00	Not done
4	15.00	8.50	4.80	+++
5	15.00	8.60	4.80	+++
6	14.75	8.80	5.20	+++
7	14.30	9.20	5.40	±
8	14.80	9.00	5.32	±
9	14.78	8.80	5.00	±
10	14.60	8.80	4.87	±
11	15.40	8.40	4.88	±
12	15.03	8.60	5.20	±
13	16.20	9.00	5.40	±
14	15.20	8.60	6.35	±
15	15.30	8.70	6.00	±
16	16.00	8.60	6.12	±
17	15.00	8.50	5.00	±
18	16.00	8.50	5.00	±
19	15.00	9.00	4.89	Not done
20	15.00	9.20	5.40	Not done
21	14.88	9.10	5.00	Not done
22	14.90	8.88	5.10	Not done
23	14.60	8.70	4.93	Not done
24	15.02	8.00	5.20	Not done
25	15.40	8.04	5.10	Not done
26	15.70	7.93	4.80	Not done
27	15.50	8.40	4.20	Not done
28	15.50	8.00	4.50	Not done
29	9.67	7.30	4.30	Not done
30	7.33	6.3	3.8	Not done
Median (mg/g)	15	8.6	5	
Mean (mg/g)	14.10	8.52	5.09	
SD	3.01	0.58	0.53	

## Discussion

Majority of the samples of SSDs collected in the study contained different phytoestrogens (daidzein, genistein and formononetin) in quantities that could be potentially detrimental to the growth and development of the embryo.

Phytoestrogens are plant derived edible items which show estrogen-like properties and are now recognized as endocrine disruptor compounds (EDCs). They can act as estrogenic agonists or antagonists capable of interfering with the synthesis, secretion and elimination of natural hormones in the body [14, 15]. Studies indicate that human embryos and foetuses are exposed to phyestrogens and testosterone when mothers consume them during pregnancy [16–18]. Phytoestrogenic substances have shown to cross placental barrier and exposure of the embryo and foetus is directly related to maternal serum circulating levels of phytoestrogen [18–21]. Also, it is suggested that metabolic and/or excretion rates of phytoestrogens are different between mother and foetus and once phytoestrogens are transferred to the embryo and foetus, they tend to stay in foetal circulation longer than maternal circulation [21]. Animal studies indicate that testosterone too can cross the placental barrier [22–24].

It is extremely difficult to examine the effects of phytoestrogens or testosterone on human development and reproduction for ethical reasons. Evidence for concern emerges from laboratory based animal studies such as on rodents and to a lesser extent in primates. The observed effects can reliably predict what is likely to happen in humans [14].

Organogenesis occurs during 5–8 weeks of gestation in humans. Any insult during this period (including intake of phytoestrogens) can result in congenital malformations [25]. Sexual differentiation occurs between week 5 and 19 of pregnancy. Evidence on its probable effect can be extrapolated from studies in rodents. In them, sexual differentiation occurs from gestational days 12–20 [26]. Despite the differences in the periods when such alterations affect reproductive and brain structures and functions, the outcomes are largely consistent across species [27]. SSDs are consumed during 6–10 weeks of pregnancy which is a very critical period during the development of a foetus.

Although phytoestrogens mimic the effects of estradiol, their effects can produce estrogenic or anti-estrogenic effects depending on the phytoestrogen-estrogen ratio in the body. This partly explains why estrogenic effects predominate in livestock, whose estradiol plasma concentrations are relatively low. In contrast, anti-estrogenic effects are reported mainly in humans in which estrogen plasma levels are relatively high [28].

An adult woman can regulate the level of endogenous hormone production even when she is exposed to an exogenous phytoestrogen because of a mature hypothalamic pituitary gonadal (HPG) axis [29]. In embryonic life, when the reproductive system is developing, androgenic and estrogenic hormones can exert ‘organizational’ effects that permanently shape the reproductive system and its function. Thereafter, adjustments to the exposure of exogenous hormones cannot be made and it can lead to adverse effects of target organs. Secondly, inappropriate exposures can permanently alter the sensitivity to hormonal signals and alter the function of HPG axis across the lifespan. During puberty and adulthood, the same hormones exert ‘activational’ and ‘functional’ effects on a ‘pre-formed’ reproductive system [14, 26].

Early exposure of female embryo and foetus to phytoestrogens has also been associated with premature thelarche, and breast cancer risks [30]. Acceleration of vaginal opening, altered oestrous cycles thereby advancing pubertal onset, a decline in fertility are some of the effects reported from exposure of animals to phytoestrogens during neonatal period [14, 31, 32]. On the other hand, exposure to testosterone can masculinize female offsprings [33–37]. What is important to note here is that even a single episode of exposure during this critical period can be harmful [27]. Early exposure of a male embryo and foetus to phytoestrogens disrupts the balance between androgen and estrogen and can impact the degree of masculinization of male foetus [18, 26]. The more severe the interference, the more severe is the manifestation, with complete lack of masculinization (i.e. genotypic male with female genitalia) representing the most extreme consequence whereas hypospadias and cryptorchidism represent progressively milder consequences [38–41]. Animal experiments have shown that all testicular dysgenesis syndrome except testicular cancer can be induced by exposure of embryo to anti androgenic compounds [42].

Exposure of a male embryo and foetus to testosterone can negatively impact the reproductive development in terms of reduced sperm count and motility [34, 43, 44]. A study on guinea pigs also highlighted that excess of testosterone during pregnancy can produce hermaphrodites [45]. Prenatal testosterone also leads to infertility, obesity, intrauterine growth retardation and insulin resistance in adulthood [46–48]. With less interference, no obvious consequences may be evident at birth and may manifest only in adulthood.

Although sexual differentiation occurs in-utero among humans and rodents, sexual differentiation of the brain (which is dependent on androgens and estrogens) occurs in-utero in humans (between 12 and 24 weeks), but during the neonatal period in rodents (few days before birth till 10 days after birth) [49]. Early sexual differentiation of the developing brain is vulnerable to endocrine disruption by phytoestrogens which modify the organization of sexually differentiated neural pathways.

Testosterone and estradiol serve as the most important factors to establish permanent sex differences in brain organization [50]. The size of the sexually dimorphic nucleus of the pre-optic area (SDN) of the brain is larger in males than females and this is largely associated with sexual partner preference [18]. Exposure of male rats to resveratrol (estrogen) during neonatal period has been shown to be associated with smaller size of SDN and reduced socio-sexual behaviour in adulthood. Exposure to testosterone is also known to suppress female behaviour of the foetus in rats [27, 33, 36, 51].

Effects of endocrine disruption in the developing foetus are likely to be subtle and not readily apparent at birth, a lesson learnt from the disastrous case of DES (diethylstilbestrol). DES, a synthetic estrogen prescribed to prevent miscarriage ultimately turned out to be a cause of vaginal dysplasia, cervical adenosis and malformations of uterus and cervix. It is therefore important to consider the ‘critical windows of exposure’ when attempting to predict potential consequences of human exposure to endocrine disruptors like phytoestrogens [52–54].

The findings of the laboratory tests warrant further detailed analyses of larger numbers of samples and animal studies to explore the possible toxic effects of these components. Only a small number of samples were available for the analyses; a larger number with representation of more areas across North India, from different sources would have benefitted the results and their implications on the harmful practice of consumption of SSDs. Also, the analyses were done to test for presence of phytoestrogens and testosterone only, owing to their known effect on the development of the embryo. However, there might be other components in the drugs that might have had an effect on the embryonic development and growth; these could not be analysed due to logistic and financial constraints. Despite the limitations of the study, the results of the analyses proving the presence of fairly large proportions of phytoestrogens and testosterone in the drugs substantiate the need to curb this harmful practice for the benefit of the society at large. The results obtained from the analyses are reliable since the analyses were conducted by an experienced professional, using standardized methods at a reputed laboratory in North India.

Intake of SSD is a harmful practice quite prevalent in North India. It has been observed largely that these drugs are prescribed by faith healers and illegal practitioners largely, who knowingly or unknowingly, engage in not only an unethical, but a very risky procedure for the patients and the growing embryo. It is imperative that this dangerous practice be relinquished at the earliest. The availability of such drugs in local chemist shops, and with faith healers can be monitored through rigorous checks with the involvement of sectors other than health.

Banning the supply of these drugs will only solve part of the problem. In Indian communities, there are certain deep-rooted cultural and social customs which have evolved over the years. Hindu scriptures mention that if a son sets fire to the funeral pyre for his parents, they would be released from the travails of this world and their soul would enter heaven. The dowry system of giving gifts to the daughter and her in-law’s family at the time of her marriage, still prevalent in many parts of the country, is another social tradition [3]. These traditions and rituals are considered as normal practice and behaviour, making them that much more difficult to curb. Traditions like these increase the demand for a male child among these communities. It is therefore prudent to bring about a positive change in the attitudes and behaviours of the community towards the female child. Various innovative schemes have been launched at the Central and State levels to highlight this important social issue and provide more support to a girl child. More needs to be done for long term gains at the community level. Making the implementation of PCPNDT Act more stringent at all levels, with punitive action taken against those who violate this law within the public as well as private health systems can be one of the steps in this direction. Behaviour change communication programs should be planned and implemented at the community level, targeting families, to change their mind-set regarding these traditions and practices. Although this will take time to be widely accepted, in the long run these interventions will help in stopping female foeticide, and in improving the status of female children in Indian communities.

## Conclusion

Son preference is a phenomenon that we are grappling with in India. SSDs are consumed during the first trimester of pregnancy to have a male baby. Consumption of such drugs is dangerous and detrimental to the growth and development of the embryo since these contain phytoestrogens and testosterone in amounts beyond the acceptable quantities. This is likely to have long term implications on the health of the community. It is important to inform the community of the harms that these practices can cause in the long run.

## Abbreviations

DES: Diethylstilbestrol
EDC: Endocrine disruptor compounds
HPG: Hypothalamic pituitary gonadal
HPLC: High performance liquid chromatography
PCPNDT: Pre conception, pre natal diagnostic technique
PNDT: Pre natal diagnostic technique
SDN: Sexually dimorphic nucleus
SSD: Sex selection drugs
TLC: Thin layer chromatography
